# Footpad Monitoring: Reliability of an Automated System to Assess Footpad Dermatitis in Turkeys (*Meleagris gallopavo*) During Slaughter

**DOI:** 10.3389/fvets.2022.888503

**Published:** 2022-05-17

**Authors:** Jenny Stracke, Robby Andersson, Nina Volkmann, Birgit Spindler, Jan Schulte-Landwehr, Ronald Günther, Nicole Kemper

**Affiliations:** ^1^Institute of Animal Science, Ethology, University of Bonn, Bonn, Germany; ^2^Institute for Animal Hygiene, Animal Welfare and Farm Animal Behavior, University of Veterinary Medicine Hannover, Foundation, Hannover, Germany; ^3^Faculty of Agricultural Sciences and Landscape Architecture, University of Applied Sciences Osnabrück, Osnabrück, Germany; ^4^Science and Innovation for Sustainable Poultry Production (WING), University of Veterinary Medicine Hannover, Foundation, Vechta, Germany; ^5^CLK GmbH, Bildverarbeitung and Robotik, Altenberge, Germany; ^6^Heidemark Mästerkreis GmbH u. Co. KG, Haldensleben, Germany

**Keywords:** footpad dermatitis (FPD), animal welfare, automatic assessment, welfare indicator, turkeys, abattoir

## Abstract

Footpad dermatitis (FPD) is an indicator of animal welfare in turkeys, giving evidence of the animals' physical integrity and providing information on husbandry management. Automated systems for assessing FPD at slaughter can present a useful tool for objective data collection. However, using automated systems requires that they reliably assess the incidence. In this study, the feet of turkeys were scored for FPD by both an automated camera system and a human observer, using a five-scale score. The observer reliability between both was calculated (Krippendorff's alpha). The results were not acceptable, with an agreement coefficient of 0.44 in the initial situation. Therefore, pictures of 3,000 feet scored by the automated system were evaluated systematically to detect deficiencies. The reference area (metatarsal footpad) was not detected correctly in 55.0% of the feet, and false detections of the alteration on the footpad (FPD) were found in 32.9% of the feet. In 41.3% of the feet, the foot was not presented straight to the camera. According to these results, the algorithm of the automated system was modified, aiming to improve color detection and the distinction of the metatarsal footpad from the background. Pictures of the feet, now scored by the modified algorithm, were evaluated again. Observer reliability could be improved (Krippendorff's alpha = 0.61). However, detection of the metatarsal footpad (50.9% incorrect detections) and alterations (27.0% incorrect detections) remained a problem. We found that the performance of the camera system was affected by the angle at which the foot was presented to the camera (skew/straight; *p* < 0.05). Furthermore, the laterality of the foot (left/right) was found to have a significant effect (*p* < 0.001). We propose that the latter depends on the slaughter process. This study also highlights a high variability in observer reliability of human observers. Depending on the respective target parameter, the reliability coefficient (Krippendorff's alpha) ranged from 0.21 to 0.82. This stresses the importance of finding an objective alternative. Therefore, it was concluded that the automated detection system could be appropriate to reliably assess FPD at the slaughterhouse. However, there is still room to improve the existing method, especially when using FPD as a welfare indicator.

## Introduction

Farm animal welfare is of increasing importance in the public perception of livestock production (1). Consequently, there is a need to find adequate methods for assessing and documenting farm animal welfare status (2). One approach is to monitor the welfare directly on-farm, with much effort taken in recent years to develop adequate assessment protocols (3, 4). Monitoring the welfare state on-farm has the benefit of enabling a direct response to potential irregularities. However, data acquisition can be time-consuming and only allows for the inspection of a fraction of the whole flock. An alternative can be to collect data at the slaughterhouse, which is both practical and feasible when scoring large numbers of animals (5). In Europe, such post-mortem inspections are prescribed by law for broilers and include monitoring abnormal levels of contact dermatitis, parasitism, and systemic illness (6). The parameters which are recorded in detail depend on local legislation and consumer demands. In fattening poultry, one important animal welfare parameter is footpad dermatitis (FPD), described as a contact dermatitis of the plantar surface of birds' feet (7). It occurs with different severity grades and can affect the surface and subjacent structures like the stratum intermedium, the stratum basale, or the dermis (7, 8). Monitoring FPD is a requirement for broilers in Sweden, Denmark, Finland, the UNITED KINGDOM, Germany, and the Netherlands (9) and countries exporting to Germany (e.g., Poland, Italy). According to the European Commission (10), 18 states request the recording of FPD by national law.

In turkeys, there are no legal standards to monitor FPD yet (11), despite that Hocking et al. (12) stated that legal regulations would occur in the future due to the apparent incidents of FPD in turkeys worldwide [see (13–15) for examples of country-specific prevalence]. Each country has its policies, and most are based on recommendations and voluntary actions. For instance, in Germany, the “National Parameters for Voluntary Agreements for the Keeping of Turkeys” (16) serve as a guideline for turkey farming (17). Here, footpad health is a major parameter to ensure adequate animal keeping. Furthermore, quality assurance programs require the evaluation of footpad health at slaughter when slaughter capacity exceeds 500 animals per hour (18). Monitoring FPD is an accepted tool not only in Germany but all over Europe and the United States [see (19) for the United Kingdom and the United States, (20, 21) for Europe]. Watanabe et al. (22) identified FPD as one of the most relevant animal-based indicators to measure animal welfare so far. This indicator not only provides information on animal health and wellbeing, but is also associated with the husbandry system, with litter quality being the major determinant for this pathology (23–26). Therefore, even the retrospective evaluation at the slaughterhouse might impact future animal welfare.

Measuring the severity of FPD is usually based on a scoring system, with different procedures being described in the literature (19, 23, 24). However, a standardized scoring system is vital to allow comparability (essential in a scientific context but also plays a great role in international market competition). This gap was filled in 2008, when Hocking et al. (20) proposed a standard classification, which is now used most commonly all over Europe. Their system defines the severity of FPD with regard to the size of the alteration in relation to the metatarsal footpad, using five categories, ranging from 0 (unaffected) to 4 (more than half the foot pad affected). Hocking et al. (20) highlighted that a reliable and valid scoring system should be clearly defined, results should be repeatable between different classifiers, and they should be quick and easy to use. Various studies provide evidence that their scoring system fulfills all these criteria (20, 27, 28). However, the application in practice is labor-intensive and, therefore, costly (29, 30). Furthermore, assessment systems are prone to observer bias (31, 32). Therefore, human observers must be trained, and consistency between different observers must be tested regularly (30, 33).

This problem can be addressed using automated systems. Precision Livestock Farming (PLF) technologies, offering opportunities to increase the efficiency and sustainability of farming and production (34), are rapidly developing in the poultry sector worldwide (35). Image analysis seems to be a promising approach to automatically evaluate FPD at the slaughterhouse (30, 36, 37). A similar technique is implemented in German slaughterhouses for turkeys (27, 38). However, the study by Vanderhasselt et al. (30) reported a poor agreement between human observers and the automated system using a prototype produced by Meyn Food Processing Technology B.V. (Noordeinde, the Netherlands). The system could be improved remarkedly, as a study by Van Harn et al. (39) showed, reporting good agreement between the automated system and human observers, but in this study, the footpad lesion score was underestimated. The camera system used in the study of Louton et al. (37) found that in broilers, intact feet and higher scoring levels could be detected with sufficient sensitivity, whereas the sensitivity for low-level severity was deficient.

Therefore, this study aimed to evaluate the status quo reliability of the automated camera system currently used to detect FPD in turkeys in German slaughterhouses and, if possible, to improve its performance.

## Animals, Materials, and Methods

The study was conducted at one slaughterhouse in Germany, using an automated system as a standard measurement to monitor the FPD of the metatarsal footpad in turkeys. The automated camera system (CLK GmbH; Turkey Check V1.0, Altenberge, Germany) was installed as a fixed part of the slaughter line. After separating the feet from the body, it was positioned at the end of the line, taking pictures of each foot passing the camera. Feet at this stage are more or less free of dirt, due to the preceding slaughter process (e.g., after scalding, plucking, and evisceration). An integrated software, based on 2-dimensional-RGB-image analysis and processing, detects the foot by contrasting the colored foot to a blue background. The metatarsal footpad is defined by calculating the biggest inner circle from the segmented foot region and applying morphological operations to the foot shape (**Table 2**, green line). FPD is defined as discoloration on the skin (darker areas); these discolorations are detected and outlined red in the picture (**Table 2**, red line). To calculate the severity of FPD, the size of the discoloration is set concerning the size of the metatarsal footpad and then allocated to one of five scoring levels. The thresholds can be freely set - for the respective slaughterhouse, the chosen thresholds can be found in Table 1. The automated camera system scores the left foot per pair by default (defined by the company); if this foot cannot be scored for any reason (pre-defined settings by the manufacturer, e.g., two feet in the slaughter hooks, no foot in the slaughter hook), the system switches to the right foot.

**Table 1:** Scoring system for footpad dermatitis (FPD) on the metatarsal footpad adapted from Hocking et al. (20).

**Scoring level**	**Definition**	
0	Intact foot	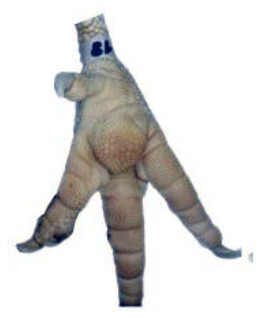
1	Small, punctual alterations covers <10% of the metatarsal footpad surface	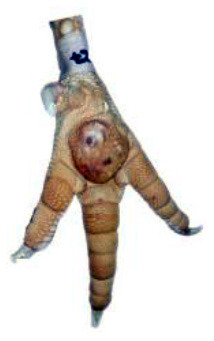
2	Altered lesion covers <25% of the metatarsal footpad surface	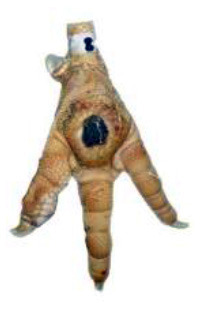
3	Altered lesion covers <50% of the metatarsal footpad surface	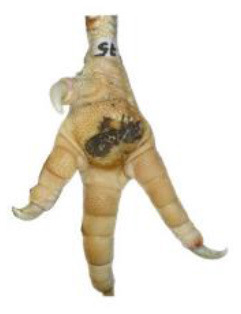
4	Altered lesion covers more than 50% of the metatarsal footpad surface	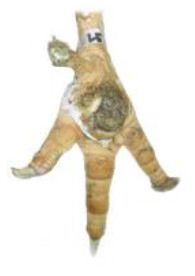

*Pictures by Jenny Stracke*.

### Scoring System and Observer Reliability

The severity of FPD was categorized using a scoring system adapted from Hocking et al. (20). The categories are described in detail in Table 1. FPD was scored by a human observer, either observing the actual tangible foot (MAN) or assessing the incidence on digital pictures produced by the automated camera system (HUM). Furthermore, the automated system scored the severity as described above (AUT). The algorithm used was either labeled AUT1 referring to the situation before modification of the automated system, or AUT2 referring to the algorithm post-modification.

Performance of the camera system (2.3 / 2.4) was evaluated by scoring the accuracy in detecting each, the metatarsal footpad, the altered area, and the presentation angle of the foot. Scoring levels and a detailed description of the scoring system can be found in Table 2.

**Table 2:** Scoring system to evaluate the accuracy of the automated camera system.

**Parameter**	**Scoring level**	**Definition**	
Metatarsal footpad	0	Detection is correct (including the gap/interspace between metatarsal footpad and toes)	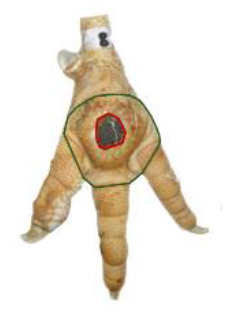
	1	Detected area is bigger than the “true” metatarsal footpad or slipped out of position; <1/2 segment of a toe	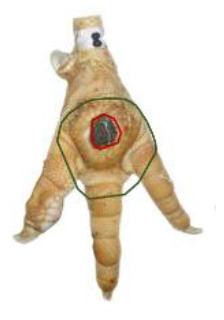
	2	Detected area is bigger than the “true” metatarsal footpad or slipped out of position; >1/2 segment of a toe	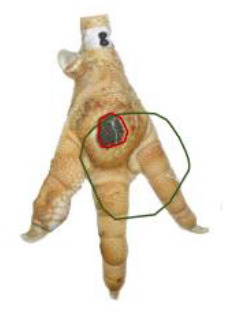
	3	Detected area is smaller than the “true” metatarsal footpad	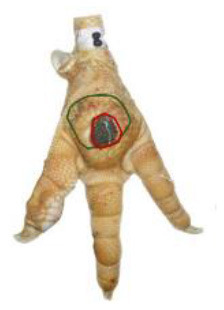
Alteration	0	Detection is correct	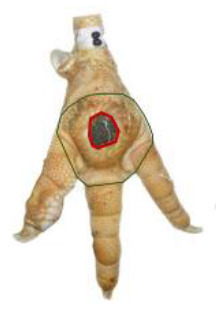
	1	Detected area is bigger than the “true” alteration	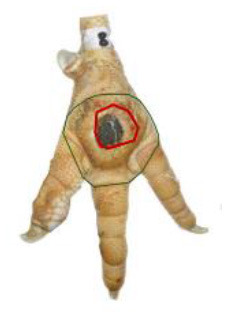
	3	Detected area is smaller than the “true” alteration	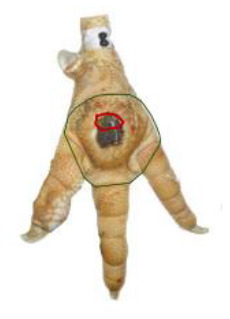
Angle of presentation	0	Aligned straight to the camera	
	1	Aligned slightly skew	
	2	Aligned skew	

“*True” metatarsal footpad refers to the definition of metatarsal footpad by the automated camera system (i.e., cutting the toes off and putting a circle around the rest; see Material and Methods for detailed description), which does not take the three-dimensional appearance of the metatarsal footpad into account. Automated detection of the metatarsal footpad is marked in green; automated detection of the alteration is marked in red; pictures by Jenny Stracke*.

Before the data acquisition started, the inter-observer reliability of human observers was calculated. For MAN, 300 pairs of turkey feet (B.U.T. 6, Aviagen Turkeys Ltd., Tattenhall, United Kingdom) of mixed sexes (200 feet of female birds and 400 feet of male birds) were sampled from the slaughter line in random order. The feet were scored by two observers. The observers (research scientists/veterinarians) were experienced in assessing FPD, observer training before data acquisition, therefore, was limited to a verbal recapitulation of the defined thresholds. To minimize the observer bias, the feet of one pair were scored separately with a time offset, first scoring the left and then the right feet.

Inter-observer reliability for HUM was calculated by scoring a subset of digital pictures produced by the automated system (HUM), which were not pre-evaluated automatically. The dataset contained 400 left and right feet of male turkeys selected from two flocks. The observers were the same as for the macroscopic scoring. Additionally, intra-observer reliability was calculated for one of the observers (main observer) on the same dataset.

For the scoring system to assess the accuracy of the automatic detection observer reliability (inter-/intra) was calculated by scoring a subset of digital pictures (*n* = 100), containing 200 left and right feet. Again both observers (research scientists) were experienced in assessing FPD, observer training, therefore, was limited to a verbal recapitulation of the scoring system.

### Initial Situation

**Figure 1:**
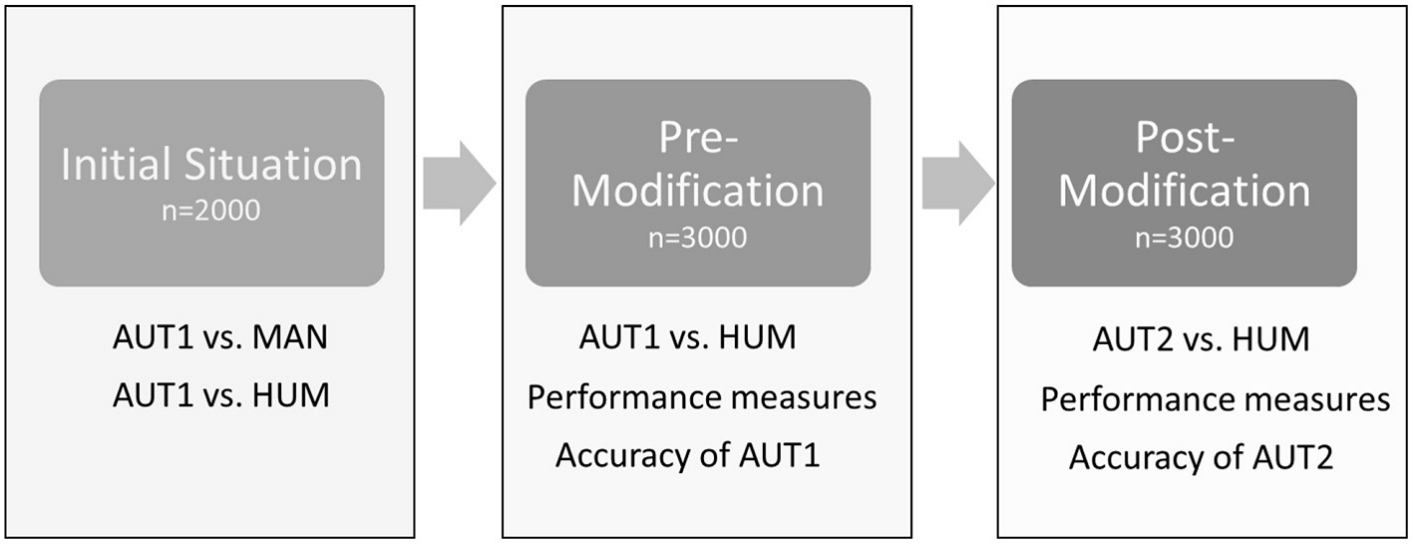
Outline of the different steps of the study. AUT1: algorithm for automated detection pre-modification; AUT2: algorithm for automated detection post-modification; MAN: scoring of footpad dermatitis by a human observer based on the actual tangible foot; HUM: scoring of footpad dermatitis by a human observer based on digital pictures produced by the automatic system.

An outline of the different steps of the study is presented in Figure 1. The initial situation was evaluated on a dataset sampled at the slaughter line in 2018 (February–April). In total, the feet of seven flocks were scored by the automated camera system (AUT1; see Table 1 for the respective scoring system), saving the pictures in the same order as feet passed the camera. Of those, in total, 2,000 feet (one foot per pair) were marked with cable straps at the beginning of the slaughter line. The feet (both feet per pair) were collected from the line after passing the automated camera system, maintaining the original order. The feet were scored macroscopically by the main observer (MAN). Digital pictures of the automated camera system were assigned to the respective score of MAN by scanning the pictures of the automated system for the feet that were identified by the cable straps (keeping the order of the pictures constant). These pictures were additionally scored by AUT1 and HUM. Of those 2,000 pictures, 19 pictures had to be excluded from further analysis, as either AUT1, MAN, or HUM was not available due to the bad quality of the pictures or data loss.

### Pre-modification

The main part of this study was done in 2019 (March–July). The automated camera system monitored three flocks of turkeys (B.U.T. 6, Aviagen Turkeys Ltd., Tattenhall, United Kingdom). A random sample of digital pictures (1,500 pairs of feet) was used as an experimental dataset. Contrary to the standard routine, both feet per pair of feet were scored automatically (AUT1). These feet were then scored by the main observer (HUM). Of the basic dataset, 354 feet were excluded due to bad image quality, which was the case if either AUT1 or HUM could not reliably score the severity grade of FPD.

The performance of the automated camera was evaluated for these feet (2,646 feet in total; 1,220 left feet; 1,426 right feet), scoring the accuracy in detecting each, metatarsal footpad, the altered area, and the presentation angle of the foot (Table 2). Further feet had to be excluded from data analysis due to bad quality in detecting either the metatarsal footpad or the alteration (seven concerning the metatarsal footpad, five concerning the altered area).

### Modification and Validation

The manufacturer modified the algorithm of the automated camera system to improve its performance. Lesion detection was set to be sharper in total but more differentiated. The segmented lesions were treated differently based on their color intensity. This allowed different heuristics to be applied in a more selective way to better detect false (pseudo) lesions.

The new algorithm (AUT2) was again run on a random set of 1,500 pairs of feet (3,000 feet) originating from the same digital data source mentioned above. Of those, 1,135 feet were already scored pre-modification, and 1,865 feet were not observed in the old dataset. Again, all feet were scored by a human observer (HUM). Altogether, 504 feet were excluded because either AUT2 or HUM could not score the severity grade of FPD reliably. For the remaining pictures (1,201 left feet, 1,309 right feet) repeating the process described in part 2.2, once more, a human observer scored the performance of the automated system for the three parameters (metatarsal footpad, altered area, angle of presentation). Again, further feet were excluded from scoring due to bad quality (14 feet concerning the metatarsal footpad, 20 feet concerning the altered area).

### Statistics

The SAS software (V.9.4, Statistical Analysis Institute, Cary, NC, USA) was used for statistical analysis.

The agreement between AUT1/AUT2, MAN, and HUM, and observer reliability was estimated by calculating the Krippendorff's alpha. This reliability coefficient does include not only perfect agreements but also considers the degree of discrepancies. More specifically: if scores between methods of measurement differ only slightly (e.g., by one scoring level), the result will turn out better compared to a situation where scoring methods differ appreciably (e.g., more than one scoring level) (40). To do so, the macro developed by Hayes and Krippendorff (41) was used, including the data type (ordinal) and set the number of bootstraps to 5,000. Each dataset was calculated separately. Observer reliability was evaluated using the classification proposed by Landis and Koch (42) (<0.00 = poor; 0.00–0.20 = slight; 0.21–0.40 = fair; 0.41–0.60 = moderate; 0.61–0.8 = substantial; 0.81–1.00 = almost perfect).

Performance measures were conducted, calculating the sensitivity, specificity, positive predicted value (PPV, precision), negative predicted value (NPV), and accuracy, setting HUM as the gold standard. Here, the NLMIXED procedure was used, fitting a Poisson model with one parameter for each cell of a 2 × 2 table and specifying the respective formula (1–5) in an ESTIMATE statement.

(1) Sensitivity = $\frac{\;\sum true\;positives}{\sum true\;positives\;+\;\sum false\;negatives}$
(2) Specificity = $\frac{\;\sum true\;negatives}{\sum true\;negatives\;+\;\sum false\;positives}$
(3) Positive predictive value = $\frac{\;\sum true\;positives}{\sum true\;positives\;+\;\sum false\;positives}$
(4) Negative predictive value = $\frac{\;\sum true\;negatives}{\sum true\;negatives\;+\;\sum false\;negatives}$
(5) Accuracy = $\begin{array}{l} \frac{\sum true\;positives+\sum true\;negatives}{\sum true\;positives+\sum true\;negatives+\sum false\;positives+\sum false\;negatives} \end{array}$

The GENMOD procedure was used to analyze the accuracy in detecting the metatarsal footpad and the accuracy of detecting the altered area, including the version of the algorithm (AUT1/AUT2), the presentation angle of the foot (scoring level 1/2+3), and the laterality of the scored foot (left/right) as a predictor. In a further model using the GENMOD procedure, the laterality (left/right) and version of the algorithm (AUT1/AUT2) were used as a predictor for the angle of the foot. The distribution was set to multinomial in each model, specifying the respective link function to cumprobit. Repeated measurements were accounted for using the repeated statement (number of the picture analyzed).

## Results

### Observer Reliability and Reliability of the Initial State

The inter-reliability between two human observers resulted in a Krippendorff's alpha of 0.82 for MAN and 0.77 for HUM. The calculation of the intra-reliability for HUM resulted in a Krippendorff's alpha of 0.85. According to the classification of Landis and Koch (42), these results can be considered almost perfect and substantial.

The inter-observer reliability for the scoring system to evaluate the accuracy of the automated camera system resulted in a Krippendorff's alpha of 0.42 for the detection of the metatarsal footpad, 0.21 for the detection of the alteration, and 0.35 for the angle the foot was presented to the camera, which can be considered as fair to moderate. The intra-observer reliability resulted in values of 0.43 (alteration) to 0.65 and 0.67 (metatarsal footpad and angle), which can be considered moderate to substantial.

Comparing the AUT1 and MAN resulted in a Krippendorff's alpha of 0.23; for the agreement between AUT1 and HUM in the initial situation, a Krippendorff's alpha of 0.44 was calculated. According to the classification of Landis and Koch (42), these values can be considered fair.

### Pre-modification

For the total agreement between AUT1 and HUM, a Krippendorff's alpha of 0.43 was calculated, considered as a fair agreement.

The detailed values for the agreements are presented in Table 3A. Most deviations in the scoring of AUT1 in comparison to HUM could be found for scoring level 1 (16.7% of the analyzed feet) and scoring level 0 (10.1% of the analyzed feet), whereas, in scoring level 0, 8.4% of the analyzed feet differed more than one scoring level (0.5% of the cases for scoring level 1). For scoring level 2 and level 3, the analyzed feet were scored differently by the AUT1 compared to HUM in 9.1 and 9.6%, respectively, with a total of 0.7% deviating by more than one scoring level. The least deviations were found for scoring level 4 (0.2% of the analyzed feet, 0.1% more than one scoring level).

**Table 3:** Measures of agreement/disagreement between scoring methods. (A): Pre-modification, (B): Post-modification.

**(A) Pre-modification**	**AUT1**					
**HUM**	Score 0	Score 1	Score 2	Score 3	Score 4	Total
Score 0	20 (0.76)	4 (0.15)	2 (0.08)	0	0	26 (0.98)
Score 1	49 (1.85)	109 (4.12)	17 (0.64)	7 (0.26)	0	182 (6.88)
Score 2	114 (4.31)	426 (16.10)	856 (32.35)	155 (5.86)	2 (0.08)	1553 (58.69)
Score 3	71 (2.68)	12 (0.45)	211 (7.97)	425 (16.06)	4 (0.15)	723 (27.32)
Score 4	36 (1.36)	1 (0.04)	10 (0.38)	93 (3.51)	22 (0.83)	162 (6.12)
Total	290 (10.96)	552 (20.86)	1096 (41.42)	680 (25.70)	28 (1.06)	2646 (100.00)
**(B) Post-modification**	**AUT2**					
HUM	Score 0	Score 1	Score 2	Score 3	Score 4	Total
Score 0	30 (1.20)	10 (0.40)	2 (0.08)	0	0	42 (1.67)
Score 1	44 (1.75)	200 (7.97)	24 (0.96)	5 (0.20)	2 (0.08)	275 (10.96)
Score 2	48 (1.91)	431 (17.17)	1029 (41.00)	144 (5.74)	5 (0.20)	1657 (66.02)
Score 3	2 (0.08)	13 (0.52)	101 (4.02)	364 (14.50)	5 (0.20)	485 (19.32)
Score 4	1 (0.04)	2 (0.08)	2 (0.08)	28 (1.12)	18 (0.72)	51 (2.03)
Total	125 (4.98)	656 (26.14)	1158 (46.14)	541 (21.55)	30 (1.20)	2510 (100.00)

*AUT, scoring of the automated system; HUM, digital pictures scored by a human observer; column marked in light gray: (perfect) agreement, column marked in gray with stripes: deviation of one scoring level, column marked in dark gray: deviation of more than one scoring level; values are presented as total numbers (left) and percent (right)*.

Performance measures reflect this result with a calculated sensitivity of 0.07 for scoring level 0. Sensitivity for the other scoring levels ranged between 0.59 (score 1) and 0.79 (score 4). Specificity was moderate to high for all scoring levels ranging between 0.55 (score 2) and 0.99 (score 0). Accuracy ranged from 0.64 (score 2) to 0.94 (score 4). The remaining performance values can be found in Table 4A. Table 4B presents the performance values for the condensed dataset with only those feet which were scored by both algorithms (AUT1 and AUT2).

**Table 4:** Performance of the automated scoring system (A): Full dataset (pre-modification: *n* = 2,646; post-modification n = 2,510); (B): Small dataset with only those feet which were scored by both algorithms (AUT1 and AUT2), *n* = 1,135.

**(A)**		**Sensitivity**	**Specificity**	**PPV**	**NPV**	**Accuracy**
Score 0	AUT1	0.07 ± 0.02	0.99 ± 0.00	0.77 ± 0.08	0.89 ± 0.01	0.89 ± 0.01
	AUT2	0.24 ± 0.04	0.99 ± 0.00	0.71 ± 0.07	0.96 ± 0.00	0.96 ± 0.00
Score 1	AUT1	0.59 ± 0.04	0.82 ± 0.01	0.19 ± 0.02	0.97 ± 0.00	0.81 ± 0.01
	AUT2	0.30 ± 0.02	0.96 ± 0.00	0.73 ± 0.03	0.80 ± 0.01	0.79 ± 0.01
Score 2	AUT1	0.78 ± 0.01	0.55 ± 0.01	0.55 ± 0.01	0.78 ± 0.01	0.65 ± 0.01
	AUT2	0.89 ± 0.01	0.54 ± 0.01	0.62 ± 0.01	0.85 ± 0.01	0.69 ± 0.01
Score 3	AUT1	0.63 ± 0.02	0.85 ± 0.01	0.59 ± 0.02	0.87 ± 0.01	0.79 ± 0.01
	AUT2	0.67 ± 0.02	0.94 ± 0.01	0.75 ± 0.02	0.91 ± 0.01	0.88 ± 0.01
Score 4	AUT1	0.79 ± 0.08	0.95 ± 0.00	0.13 ± 0.03	0.99 ± 0.00	0.94 ± 0.01
	AUT2	0.60 ± 0.09	0.99 ± 0.00	0.35 ± 0.07	0.99 ± 0.00	0.98 ± 0.00
**(B)**		**Sensitivity**	**Specificity**	**PPV**	**NPV**	**Accuracy**
Score 0	AUT1	0.07 ± 0.02	0.99 ± 0.00	0.64 ± 0.13	0.89 ± 0.01	0.89 ± 0.01
	AUT2	0.23 ± 0.05	0.99 ± 0.00	0.64 ± 0.10	0.95 ± 0.01	0.94 ± 0.01
Score 1	AUT1	0.18 ± 0.02	0.95 ± 0.01	0.57 ± 0.05	0.77 ± 0.01	0.75 ± 0.01
	AUT2	0.34 ± 0.03	0.96 ± 0.01	0.75 ± 0.04	0.80 ± 0.01	0.79 ± 0.01
Score 2	AUT1	0.86 ± 0.02	0.46 ± 0.02	0.54 ± 0.02	0.82 ± 0.02	0.63 ± 0.01
	AUT2	0.90 ± 0.01	0.51 ± 0.02	0.62 ± 0.02	0.86 ± 0.02	0.69 ± 0.01
Score 3	AUT1	0.61 ± 0.03	0.90 ± 0.01	0.60 ± 0.03	0.90 ± 0.01	0.84 ± 0.01
	AUT2	0.58 ± 0.03	0.95 ± 0.01	0.72 ± 0.03	0.91 ± 0.01	0.88 ± 0.01
Score 4	AUT1	0.55 ± 0.15	0.97 ± 0.01	0.15 ± 0.06	0.99 ± 0.00	0.97 ± 0.01
	AUT2	0.40 ± 0.15	0.99 ± 0.00	0.21 ± 0.0	0.99 ± 0.00	0.98 ± 0.00

*AUT1: algorithm for automated detection pre-modification; AUT2: algorithm for automated detection post-modification; PPV: positive predicted value; NPV: negative predictive value; data is presented ± standard error; dark gray indicating an increase; light gray indicating a decrease*.

**Figure 2:**
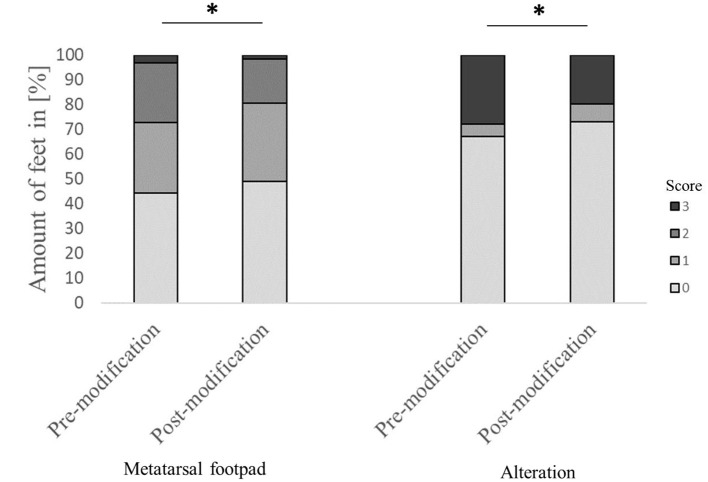
Comparison of the performance of the automated camera system in detecting the metatarsal footpad and the alteration before and after modification of the algorithm. Data are presented as the number of feet in percent for the respective scores with increasing gray levels indicating an increasing score level (light gray = scoring level 0–dark gray scoring level 3; see Table 2 for the explanation of scoring levels); *p < 0.05; the number of feet for the detection of the metatarsal footpad: pre-modification = 2,639, post-modification = 2,505; the number of feet for the detection of the alteration: pre-modification = 2,632, post-modification = 2,490.

Evaluating the accuracy of the detection in detail found that in 44.4% of the feet, the metatarsal footpad was detected perfectly well. However, in 52.2% of the analyzed feet, the metatarsal footpad was estimated as too big or slipped out of position (28.3% for score 1; 23.9% for score 2), whereas in 3.3% of the feet, the automated camera system assessed the metatarsal footpad smaller than the “true” metatarsal footpad. For the detection of the alteration, correct performance could be found in 67.1% of the analyzed feet; in 5.1% of the feet, the alteration was estimated as too big; in 27.8% of the detections, the alteration was assessed smaller as the true alteration (Figure 2). Furthermore, in 58.7% of the feet, the foot was presented straight to the camera; in 41.3% of the feet, the angle of the presentation was skewed (36.9% score 1, 4.4% score 2).

### Modification and Validation

The total agreement between AUT2 and HUM resulted in a Krippendorff's alpha of 0.62, which can be considered as a substantial agreement. The calculation of the intra-observer reliability (calculated for feet estimated before and after modification only, HUM) resulted in a Krippendorff's alpha of 0.61.

The agreement between AUT2 and HUM is presented in Table 3B. Again, most deviations were found for scoring level 1, where AUT2 scored differently than HUM in 18.2% of the analyzed feet (2.0% differing more than one scoring level). Agreement between AUT2 and HUM in the other scoring classes improved compared to the results for the pre-modification dataset, resulting in 3.8% differing classifications for scoring level 0 (2.0% differing more than one scoring level), 5.1% differing classifications for scoring class 2 (0.1% differing more than one scoring level), 7.1% differing classifications for scoring level 3 (no feet more than one scoring level), and 0.5% deviations in scoring level 4 (0.2% differing more than one scoring level).

The results of the performance measures can be found in Table 4A. Again, Table 4B presents the performance values for the condensed dataset with only those feet which were scored by both algorithms (AUT1 and AUT2). The sensitivity increased for scoring level 0 (0.24); for the other scoring classes, sensitivity ranged between 0.30 (scoring level 1) and 0.89 (scoring level 2). Specificity spanned from 0.54 (scoring level 2) to 0.99 (scoring levels 0 and 4). Accuracy improved slightly in all scoring levels except for scoring level 1 (0.79), now ranging between 0.69 (scoring level 2) and 0.98 (scoring level 4).

**Figure 3:**
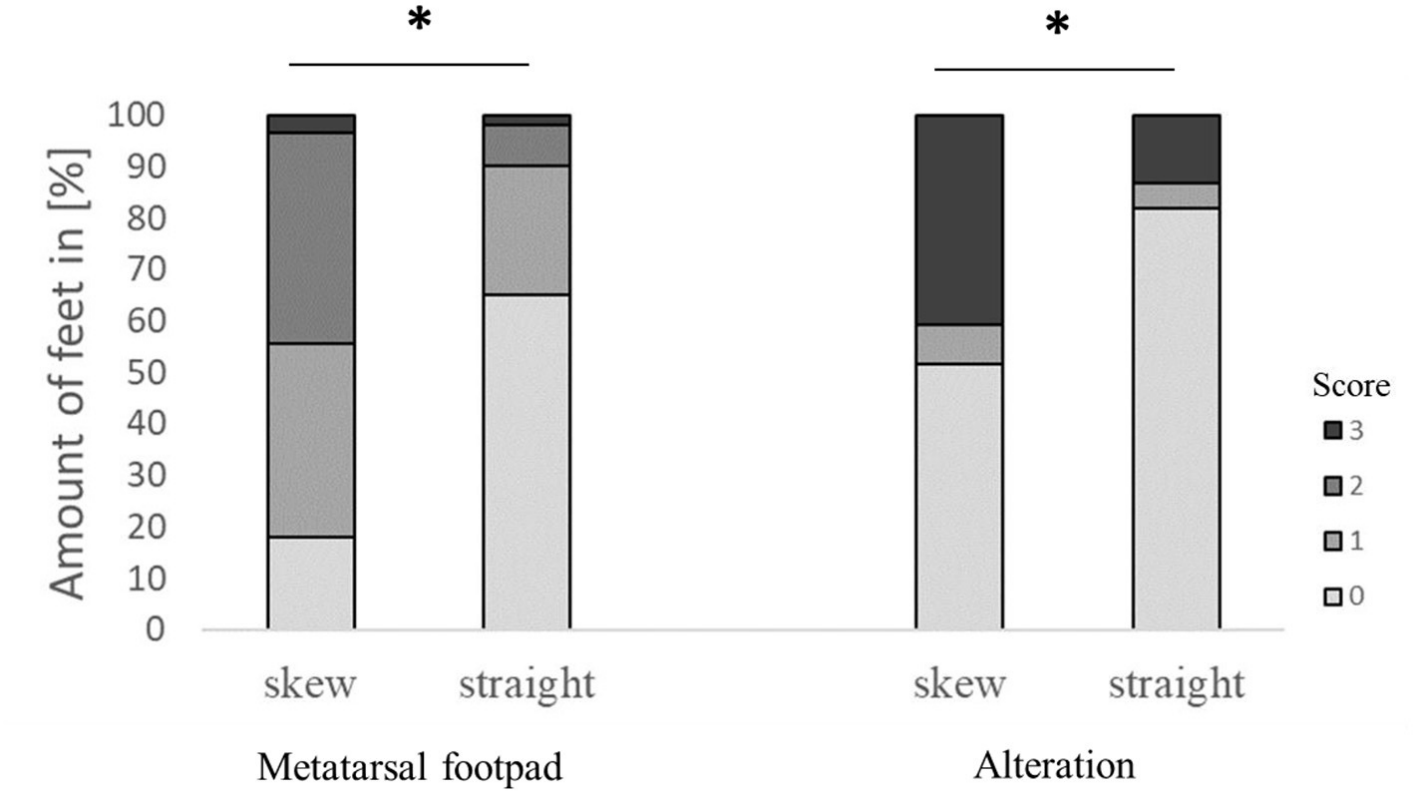
Effect of the angle the foot was presented to the camera on the performance in detecting the metatarsal footpad and the alteration. Data are presented as the number of feet in percent for the respective scores with increasing gray levels indicating an increasing score level (light gray = scoring level 0–dark gray scoring level 3; see Table 2 for the explanation of scoring levels); *p < 0.05; the number of feet for the detection of the metatarsal footpad: skew = 2,008, straight = 3,127; the number of feet for the detection of the alteration: skew = 1,996, straight = 3,119.

The modification significantly affected detecting the metatarsal footpad and the alteration (both *p* < 0.001). In 49.1% of the detections, the metatarsal footpad was identified correctly; there was a decrease in detections where the metatarsal footpad was assessed as too small (1.6%) compared to the dataset before modification. The detections where the metatarsal footpad was estimated bigger than the “true” metatarsal footpad or slipped out of position also decreased; however, the results still showed a misclassification rate of 49.3% (31.5% for scoring level 1, 17.8% for scoring level 2). For the detection of alterations, correct estimation increased to 73.0%, alterations assessed as too big slightly increased to 7.1%, whereas detections estimated smaller than the true alteration decreased to 19.7% (Figure 2). In 63.1% of the analyzed feet, the foot was presented straight to the camera, whereas the angle of the rest was skewed (34.3% for score 1, 2.6% for score 2). There was a significant difference between AUT1 and AUT2 (*p* < 0.001) for the presentation angle. The angle then again revealed a significant effect on the detection of the metatarsal footpad and the alteration (both *p* < 0.001) (Figure 3). When presented straight to the camera, the metatarsal footpad was detected correctly in 65.1% of the feet, and the alteration was determined correctly in 81.9%. In the feet where the angle was skewed, 78.6% of the metatarsal footpads were estimated as too big or slipped out of position, and 40.8% of the alterations as too small.

**Figure 4:**
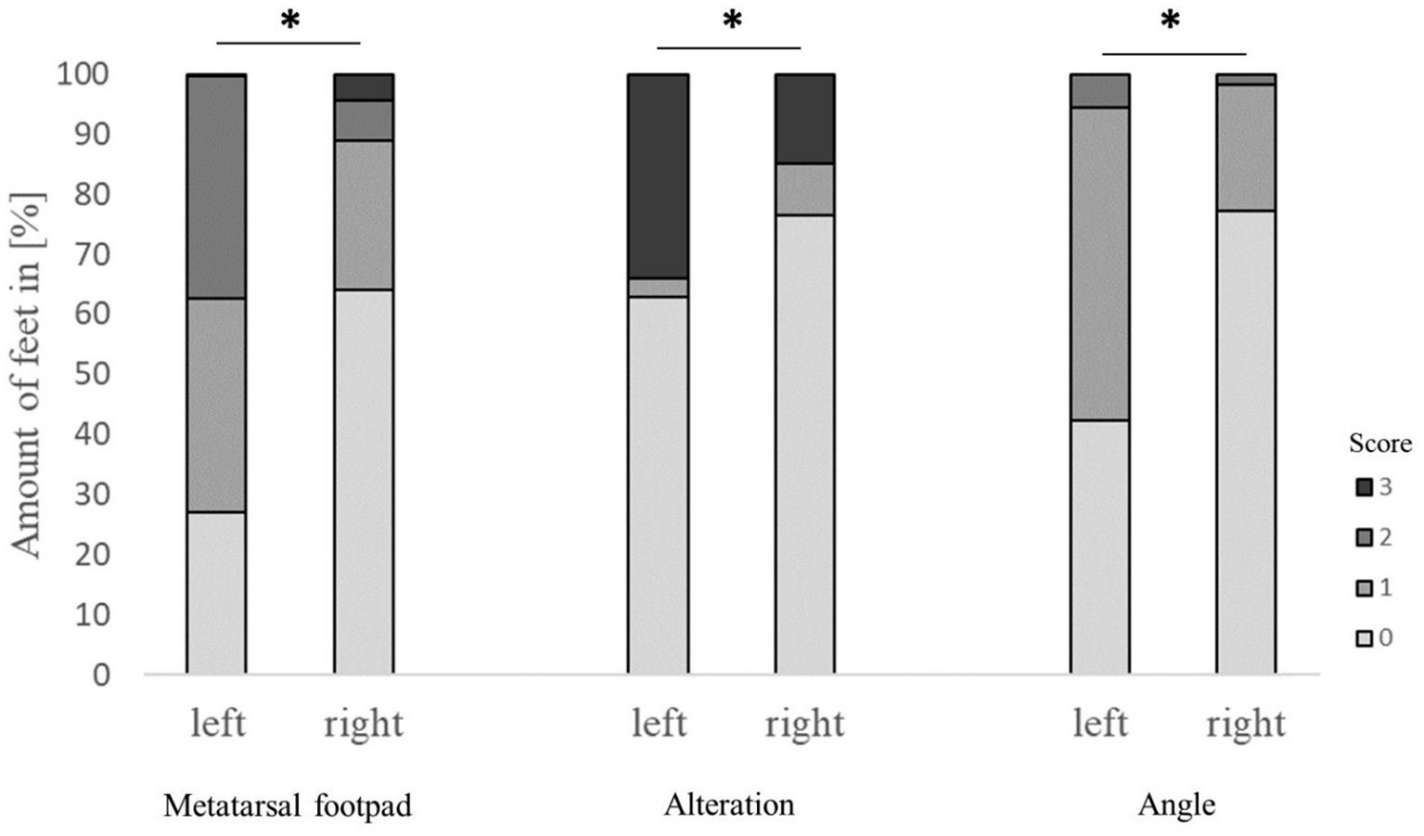
Effect of the laterality (scoring on left or right foot) on the performance in detecting the metatarsal footpad, alterations, and the angle of presentation. Data are presented as the number of feet in percent for the respective scores with increasing gray levels indicating an increasing score level (light gray = scoring level 0–dark gray scoring level 3; see Table 2 for the explanation of scoring levels); *p < 0.05; the number of feet for the detection of the metatarsal footpad: left = 2,412, right = 2,717; the number of feet for the detection of the alteration: left = 2,401, right = 2,714; the number of feet for the angle of presentation: left = 2,417, right = 2,730.

The performance of the detection of metatarsal footpad and alteration also depended on the laterality of the respective foot, finding a significant difference between the left and right feet (*p* < 0.001). Also, there was a significant difference in the presentation angle between the left and right feet (Figure 4). In the left feet, most of the metatarsal footpads were estimated as too big or slipped out of position (72.6%), whereas in the right feet, the estimation of the metatarsal was correct in most cases (64.2%). Alterations were valued too small in 33.9% of the left feet, whereas this misjudgment could be found in only 15.0% of the right feet. In the right feet, most of the feet were presented straight to the camera (77.2%), whereas in more than half of the left feet, the angle of the presentation was skewed (57.6%).

## Discussion

FPD is an accepted indicator for animal welfare in turkeys, giving evidence of the animals' health and providing information on animal husbandry management. This study aimed to evaluate the reliability of an automated camera system, detecting FPD in turkeys at the slaughterhouse, and improving its performance. Therefore, the feet of turkeys were scored for FPD by an automated camera system and a human observer, using a five-scale score.

This study could show that modifying the algorithm could improve results remarkedly concerning the agreement between a human observer and the automated camera system. The study identified several issues which remained critical for a valid automated scoring of FPD at the slaughterhouse; however, the addressed issues might be better solved by the management of the automated system rather than by changes to the algorithm of the system itself (e.g., changing the pre-defined settings; scoring of both feet).

The study was part of a large project aiming to monitor and improve the automated detection of welfare indicators in poultry. Therefore, the study started with a status quo survey, monitoring the performance of an automated camera system to measure the severity of FPD, which is already established in German slaughterhouses (18). Agreement between the results of the AUT1 and the observations by a human observer (MAN/HUM) resulted in a reliability coefficient which, according to a commonly used classification (42), could be considered a fair agreement. Two ways of human observations were included, macroscopically evaluation (MAN) and an evaluation of the digital pictures (HUM). As expected, the observer reliability was better for the agreement between AUT and HUM than the agreement of AUT and MAN, as MAN provides the possibility of a spatial perception of the foot, which is not present in the digital picture (27). According to Hocking et al. (20), scoring systems for FPD (and in general) have to fulfill multiple criteria, mainly objectivity, reliability, and repeatability. This is especially the case when using the classification as a source for an (inter-)national benchmarking as then; the classification system also has to withstand economic competition (39, 43). The criteria mentioned by Hocking et al. (20) also play a role in using classification systems to measure animal welfare, as welfare indicators are of public concern, including a mandatory need for transparent measurements (44). Concerning the automated scoring of FPD at the slaughterhouse, scoring would also need to resist the comparability with a human observer, to guarantee a valid FPD assessment even in the case of a system breakdown, and/or the comparison with slaughterhouses not using an automated system yet. With a value of 0.44, the attained reliability between automated and human-based FPD assessment was considered unsatisfactory. Therefore, the main study aimed to improve the performance of AUT1. The study performance was conducted on an experimental dataset of digital pictures; a one-to-one transmission of the results, therefore, must be treated with caution, as in practical application, other factors such as light, humidity, contamination, etc. might influence the results (37, 45). Brünger et al. (46), training neural networks for the automated detection of tail lesions of pigs at the slaughterhouse, reported issues in human observer agreement due to the quality of the digital pictures. The slight discrepancies in observer reliabilities found between human observers scoring either “real” feet (0.82) or the feet on digital pictures (0.77) might be due to such quality issues. However, this effect might also be caused by the optical bias described above (27). All pictures used were produced at the standard slaughter line during routine monitoring of FPD, using the standard default settings (e.g., threshold values), which are customary at this respective slaughterhouse.

Pre-modification, an agreement between AUT1 and HUM, with a value of 0.43, was comparable to the initial situation. Looking in greater detail, AUT1 scored feet more generous, resulting in an overestimation of scoring levels 0 and 1, whereas scoring levels 2, 3, and 4 were rated less than in the HUM. This was reflected in the performance measures, resulting in lower sensitivities in the low scoring levels.

Even if the agreement between AUT2 and HUM after the modification improved substantially (to 0.62), the performance measures did not improve equally. This can be explained by the character of the Krippendorff's alpha. As described above, the Krippendorff's alpha considers the degree of discrepancies, which means that, if the given score levels differ only slightly (e.g., by one scoring level), the result would turn out better than if the score-level difference was more pronounced (e.g., more than one scoring level) (40). Therefore, the improvement of the alpha value can be explained by less pronounced deviations, which also becomes apparent when looking at the detailed agreements for the individual scores. Calculating the performance only considers “perfect” agreements; therefore, the performance of AUT2 is still expandable concerning the results found. With a sensitivity of <0% for four of the five scoring levels, the camera system did not fulfill the criteria De Jong et al. (47) set for automated camera systems in broilers, who state that lesion class scores indicated by the software should at least display 75% agreement with a “golden standard.” However, in contrast to sensitivity, pre- and post-modification were characterized by high specificities. When using measurements in a (monetary) benchmark system, this is essential to not overestimate FPD occurrence and therefore frustrate the persons involved rather than provide incentives (48). Technically, an improvement of the sensitivity would be possible, but accompanied by a risk of a loss in specificity–therefore, finding the right balance between the performance criteria depends on how the respective interest measurements are implemented. It might be worth discussing this issue further, especially when using FPD as a welfare indicator rather than as benchmark criteria, as the focus then would benefit from a shift toward a higher sensitivity (49).

The deficiencies in scoring the low severity scores of FPD were in line with the findings for the automated assessment of FPD and hock burn lesions in broilers (37, 50). Not only for automated systems, but in human observers, the classification of less severe lesions seems to be more deviant than severe scoring classes, with Lund et al. (43) discussing a poor inter-rater agreement to be influenced by the severity of lesions. This leads to another issue that must be discussed, as performance measures and reliability scores in this study always assumed the human observation as the gold standard. There is evidence that evaluating the size of a lesion can be prone to observer bias (32, 51–53). Observing training can minimize this effect (30, 33), which can be time-consuming and costly. In this study, observers were well-trained in scoring FPD, as observer reliabilities calculated at the beginning of the study showed. Nevertheless, reproducibility of scoring results was not as good as expected, with repeated measurements of the same dataset resulting in intra-observer reliability of 0.61, which–even if considered as substantial due to the classification of Landis and Koch (42)–leaves room for improvement. The problem of human malfunctions is also known in other studies working on the automatization of diverse issues, for instance, the detection of injuries in turkeys (54) or tail lesions in pigs (46). Using an external standard as proposed by Toppel and Wernigerode (55) might be a good approach to addressing these human errors.

Improving scoring methods might not only be based on an improvement of the agreement between human and automated scoring but rather should reduce the vulnerabilities of the automated camera system itself. This study identified shortcomings in detecting the metatarsal footpad and the alteration, with the metatarsal footpad being detected as too big or slipped out of position and the alteration being estimated as too small in most of the cases. This could lead to underestimating higher severity scores by the automated camera system. However, the evaluation of the camera system's performance in detecting the metatarsal footpad and the alteration again was based on a human observer. Therefore, the problems mentioned above should be kept in mind when interpreting the results. The low inter-observer reliabilities found for the scoring method emphasize this issue, even if intra-observer reliability turned out to be substantial.

An important finding of this study was that the feet were not presented straight to the camera in many cases, which was found to harm the detection of the mentioned parameters due to a distorted perspective. The percentage of feet that presented skew to the camera decreased after modifying the algorithm. This was likely due to the different feet used pre- and post-modification rather than the algorithm itself. Interestingly, fewer feet were presented skew to the camera on the right feet than the left feet, with laterality also harming the performance in detecting metatarsal footpad and alteration. This can be explained by the procedure of the respective slaughter line. The automated camera system was installed at the end of the slaughter line after cutting the feet from the rest of the body. Cutting off the feet starts with the right foot, leaving the bodyweight hanging on the remaining (left) foot for a fraction of a second. This procedure leaves one of the feet hanging straight, while the other foot is presented skew if the camera system is adjusted to expect a “perfect position.” Therefore, the individual slaughter process must be considered when placing the camera system. In Germany, up to now, the left foot is referred to when scoring FPD–given the results of this study, this might not be the perfect solution for every company and should be considered for each slaughterhouse independently concerning the respective conditions. An update of the hardware, like the positioning system reported by van Harn and de Jong (39) for the assessment of FPD in broilers, could improve the detectability of feet in turkeys, too. The positioning system contains a panel, which works as a guide rail for the feet. Therefore, each foot is presented to the camera in the same position.

Another possibility to improve performance quality would be to ease the detection of the reference. Concerning manual FPD scoring systems, the automated systems on the market use the metatarsal footpad as a reference, which raises the question of how to define the borders properly. This is done by color detection, using the contrast between the brighter skin colors at the toes vs. the dark background color in the interspaces between the toes, eliminating the toes and putting a circle around the rest. Toppel et al. (27) discuss the correct definition of the metatarsal footpad, and the presented study results stress this question further. One possibility to circumvent this problem would be to refer to the whole foot instead, which would also offer the possibility to evaluate alterations on the toes and make the scoring of FPD more comprehensive (38). However, this would entail the difficulty of presenting the foot to the camera system in the same position with an exact orientation of the toes in each case of measurement. Otherwise, this would lead to incorrect estimations of the severity of FPD.

## Conclusion

To conclude, this study evaluated the performance of an automated camera system to detect FPD in turkeys, which is established in most German slaughterhouses. The initial situation analysis resulted in fair observer reliabilities between human observers and the automated camera system. Therefore, the algorithm of the automated system was applied to an experimental dataset of digital pictures, which were taken at the slaughterhouse. After again recording only fair agreements with a human observer and detailed failure analysis, the system was modified to improve observer reliability between the automated camera system and a human observer. Therefore, the automated detection system can be considered an appropriate approach to reliably assess FPD at the slaughterhouse. However, there is much upward scope to improve the existing method, especially concerning detecting the metatarsal footpad and the alteration. The study found that the detection quality was affected by the angle the foot presented to the camera, which also depended on the slaughter process. Thus, the quality of detection depends on individual settings and the management of the respective end-user.

## Author's Note

Footpad dermatitis (FPD) is an accepted indicator for animal welfare in turkeys, giving evidence of the animals' physical integrity and providing information on animal husbandry management. Automated systems for assessing FPD at slaughter can present a useful tool for objective data collection, with increasing importance for slaughterhouses in a rising number of countries.

Using automated systems requires them to assess the incidence reliably, especially comparability (which is essential in a scientific context and plays a great role in international market competition). Therefore, this study evaluated the status quo reliability of an automated camera system and improved its performance. The analysis of the initial situation resulted in fair observer reliabilities between human observers and the automated system, which could be improved remarkedly after modification. We found high variability in observer reliability even between human observers, considered the “gold standard,” which underlines the potential of automated systems. However, the study's data also highlight unresolved issues, and we propose that these might best be solved by defining appropriate settings and adjusting the automated system to the respective management procedure at the slaughter line.

## Data Availability Statement

The original contributions presented in the study are included in the article, further inquiries can be directed to the corresponding author/s.

## Ethics Statement

Ethical review and approval was not required for the animal study because data collection took place after slaughter, and all animals were slaughtered for use in the food chain. Therefore, no ethical approval was necessary for the animals used in this study. Slaughter procedures were in accordance with the German Animal Welfare Act and national and international regulations on the welfare of animals at slaughter [Council Regulation (EC) No 1099/2009, of 24 September 2009, on the protection of animals at the time of killing].

## Author Contributions

RA, BS, RG, JS-L, and NK were responsible for the financial acquisition and project development. JS, RA, BS, RG, JS-L, and NK contributed to the conception and design of this study. JS and BS were the principal investigators for this project. JS, RG, and NV participated in data collection. JS designed and performed the statistical analysis, contributed figures, tables, and wrote the first draft of the manuscript. JS, NV, and JS-L performed the data analysis. All authors were involved in interpreting the results, contributed to manuscript revision, read, and approved the submitted version.

## Funding

The study was conducted within the project Autowohl–Automatic detection of animal welfare indicators (FKZ: 2817903515). The project was supported by funds from the Federal Ministry of Food and Agriculture (BMEL) based on a decision of the Parliament of the Federal Republic of Germany *via* the Federal Office for Agriculture and Food (BLE) under the innovation support program. The publication was supported by Deutsche Forschungsgemeinschaft, a German research funding organization, and the University of Veterinary Medicine Hannover, Foundation, Germany, within the funding program Open Access Publishing.

## Conflict of Interest

RG was employed by Heidemark Mästerkreis GmbH u. Co. JS-L was employed by CLK GmbH. The remaining authors declare that the research was conducted in the absence of any commercial or financial relationships that could be construed as a potential conflict of interest.

## Publisher's Note

All claims expressed in this article are solely those of the authors and do not necessarily represent those of their affiliated organizations, or those of the publisher, the editors and the reviewers. Any product that may be evaluated in this article, or claim that may be made by its manufacturer, is not guaranteed or endorsed by the publisher.
